# Subtype classification based on t cell proliferation-related regulator genes and risk model for predicting outcomes of lung adenocarcinoma

**DOI:** 10.3389/fimmu.2023.1148483

**Published:** 2023-04-03

**Authors:** Qin Yang, Weiyuan Zhu, Han Gong

**Affiliations:** ^1^ School of Basic Medicine, Shaoyang University, the First Affiliated Hospital of Shaoyang University, Shaoyang, Hunan, China; ^2^ Molecular Biology Research Center and Center for Medical Genetics, School of Life Sciences, Central South University, Changsha, Hunan, China

**Keywords:** immunity, lung adenocarcinoma, mutation, predictive risk model, T cell proliferation-related regulator genes, tumor microenvironment

## Abstract

**Background:**

Lung adenocarcinoma (LUAD), the major lung cancer histotype, represents 40% lung cancers. Currently, outcomes are remarkably different in LUAD patients with similar AJCC/UICC-TNM features. T cell proliferation-related regulator genes (TPRGs) relate to the proliferation, activity and function of T cells and tumor progression. The values of TPRGs in classifying LUAD patients and predicting outcomes remain unknown.

**Methods:**

Gene expression profile and corresponding clinical data were downloaded from TCGA and the GEO databases. We systematically analyzed the expression profile characteristics of 35 TPRGs in LUAD patients and investigated the differences in overall survival (OS), biology pathway, immunity and somatic mutation between different TPRGs-related subtypes. Subsequently, we constructed a TPRGs-related risk model in TCGA cohort to quantify risk scores using LASSO cox regression analysis and then validated this risk model in two GEO cohorts. LUAD patients were divided into high- and low-risk subtypes according to the median risk score. We systematically compared the biology pathway, immunity, somatic mutation and drug susceptibility between the two risk subtypes. Finally, we validate biological functions of two TPRGs-encoded proteins (DCLRE1B and HOMER1) in LUAD cells A549.

**Results:**

We identified different TPRGs-related subtypes (including cluster 1/cluster A and its counterpart cluster 2/cluster B). Compared to the cluster 1/cluster A subtype, cluster 2/cluster B subtype tended to have a prominent survival advantage with an immunosuppressive microenvironment and a higher somatic mutation frequency. Then, we constructed a TPRGs-related 6-gene risk model. The high-risk subtype characterized by higher somatic mutation frequency and lower immunotherapy response had a worse prognosis. This risk model was an independent prognostic factor and showed to be reliable and accurate for LUAD classification. Furthermore, subtypes with different risk scores were significantly associated with drug sensitivity. DCLRE1B and HOMER1 suppressed cell proliferation, migration and invasion in LUAD cells A549, which was in line with their prognostic values.

**Conclusion:**

We construed a novel stratification model of LUAD based on TPRGs, which can accurately and reliably predict the prognosis and might be used as a predictive tool for LUAD patients.

## 1 Introduction

Lung cancer is the world’s leading cause of cancer death. Non-small cell lung cancer (NSCLC) comprises 85% of all lung cancers. Lung adenocarcinoma (LUAD) is the major NSCLC histotype, which accounts for 40% of all lung cancers (1). Due to highly heterogeneous nature and wide range of mutations, LUAD treatment is still particularly challenging. Targeted therapies that inhibit multiple oncogenic drivers and immune checkpoints have showed promise for the treatments of lung cancer, particularly LUAD, in recent years (2, 3). Currently, traditional AJCC/UICC-TNM stratification systems are the mainstay clinical determinants of the prognosis of LUAD prognosis. However, outcomes are remarkably different in patients with similar AJCC/UICC-TNM features after receiving the same treatments. To choose the best therapy for individual patient, we still need prognostic models that can better classify LUAD patients based on the likely outcome.

The reasons for outcomes are embedded into tumor tissue and complex interactions between tumor tissue and tumor microenvironment (TME) (4, 5). In NSCLC TME, T cells dominate immune cell infiltrates (6). Upon recognition of antigens, T cells proliferate and acquire capacity to kill tumor cells and secrete cytokines to coordinate the immune response. T cell proliferation modulates TME by affecting the clustering and T cell population. T cell proliferation has long been used as a tumor-reactivity marker and is positively associated with outcomes of immune checkpoints inhibitors (7–9). However, abundant evidence argues that T cell proliferation is imperfect for measuring tumor-reactivity and outcomes (8, 10–15). Recently, Mateusz Legut et al., for the first time, defined T cell proliferation-related regulator genes (TPRGs) (16, 17). In that article, many positive TPRGs that enhance T cell functions are identified (17). Therefore, comprehensive analysis of the molecular characteristics and clinical relevance in TPRGs and their relationships with TME will enhance understanding of TPRGs and improve anti-tumor strategies.

In this study, we investigate prognostic value of TPRGs and identify TPRGs-related subtypes in LUAD cohort from The Cancer Genome Atlas (TCGA). Our findings reveal that the TPRGs-related subtypes have obviously different clinical prognosis and characteristics. Additionally, we establish and validate a 6-gene risk model for predicting the overall survival (OS) based on differentially expressed genes (DEGs) between TPRGs-related subtypes. We also systematically compare the differences (including biology function and pathway, somatic mutation, immunity and drug susceptibility) between patients of different subtypes. At last, we validate biological functions of two TPRGs-encoded proteins (HOMER 1 and DCLRE1B) in LUAD cells A549. Our results show that TPRGs play an essential role in tumor progression and lays a foundation for implementing rational intervention strategies in cancer.

## 2 Materials and methods

### 2.1 Dataset acquisition

By conducting the R package “TCGAbiolinks” (18), we obtained the gene expression matrix of TCGA-LUAD (including 524 tumor and 58 normal specimens) and corresponding clinicopathological data. Detailed, the workflow type was set to “STAR-Counts” form and then expression matrix was collated as the “FKPM” format. Patients with survival time less than 1 month were excluded from subsequent analyses. Simple nucleotide variations data in format of “maf” were retrieved from TCGA portal (TCGA-LUAD project) and copy number variation (CNV) data were obtained from the term “GDC TCGA-LUAD” of UCSC Xena website (http://xena.ucsc.edu/). We obtained two external microarray datasets GSE31210 (including 246 LUAD patients and survival imformation) and GSE68465 (including 442 LUAD patients and survival imformation) from the GEO database. A total of 35 TPRGs were extracted from the published studies (16).

### 2.2 Identification of the prognostic genes and TPRGs-related clusters

We used the univariate Cox regression method to screen TPRGs with prognostic value and obtained 5 TPRGs. Based on the expression (log2 transform) of 5 TPRGs, we performed the R package “Consensus-ClusterPlus” (19) to classify LUAD patients into two TRPG-related subtypes, with the arguments of clusterAlg = “km” and distance = “euclidean”. We conducted the R package “factoextra” to perform the PCA algorithm for visualizing the distribution of two TRPG-related subtypes.

### 2.3 Biological processes quantification

We performed the R package “Gene set variation analysis (GSVA)” (20) to assess the enrichment scores of three gene sets for each LUAD patient (including v2022.1 versions of HALLMARK, KEGG, and GO-BP) from the MSigDB database (downloaded on 17 November 2022). We performed the limma package to investigate the differences in the pathway activity of distinct subtypes.

### 2.4 TME infiltration and genomic alteration analysis

The infiltration scores of TCGA-LUAD patients were retrieved from the ImmuCellAI online tool (http://bioinfo.life.hust.edu.cn/ImmuCellAI#!/) (21). Besides, we conducted the R package “estimate” to evaluate the scores of TME infiltration (including immune, stromal, and ESTIMATE score) in each subtype (22). We utilized the CIBERSORT approach to evaluate the infiltration proportions of 22 immune cells in each LUAD patient (23). We performed the maftools package (24) to read the “maf” file of LUAD patients and compare the incidence of somatic mutations between distinct LUAD subtypes.

### 2.5 Gene clustering based on DEGs of TPRGs-related subtypes

After deletion of genes that were lowly expressed in at least half of the LUAD patients (FPKM< 1), we utilized the R package “limma” to explore dysregulated genes between two TPRGs-related subtypes with |log2FC| >1 and p-adjust< 0.05 as thresholds. Consistent with previous method, we utilized consensus clustering algorithm to identify LUAD patients into two gene subtypes.

### 2.6 Construction and validation of a 6-gene risk model for LUAD patients

To evaluate the survival time of each LUAD patient, we first performed the univariate Cox regression analysis on the dysregulated genes to obtain DEGs with prognostic value. Then, least absolute shrinkage and selection operator (LASSO) cox was executed to generate a risk model, with the arguments of 10-fold cross-validation and 1000 permutations. The final risk score of each LUAD patient was defined based on the following specific formula: $Risk\;score=\sum_{i=1}^{n}Coef_{i}\;\times Exp_{i}$ , which i means one of 6 genes (CCNA2, HMMR ANLN, NKX2-1, SFTPB and KRT6A). Then, our team divided LUAD patients into two subgroups (high-risk and low-risk) according to the median risk score. We used previous two external cohorts to validate the robustness of this risk model and conducted the receiver operating characteristic (ROC) curve to evaluate the performance. In addition, we combined univariate and multivariate Cox analyses to validate the independence of this risk model.

### 2.7 Prediction of immunotherapy response and chemotherapy susceptibility

Tumor immune dysfunction and exclusion (TIDE) was an online tool to predict patient immunotherapy response based on immune-related biomarkers (25). By uploading transcriptional profiles of LUAD patients to TIDE, we obtained TIDE scores and the reaction to immunotherapy of each LUAD patient. By performing the R package “oncoPredict”, we estimated the sensitivity of about 200 drugs for each LUAD patient based on pharmacogenomic data of genomics of drug sensitivity in cancer (GDSC) 2 database as training dataset (26).

### 2.8 Cell lines and cell culture

LUAD cell line A549 was purchased from the Cell Bank of Type Culture Collection of the Chinese Academy of Sciences, Shanghai Institute of Cell Biology. A549 cells were grown in RPMI 1640 medium (Gibco, United States) supplemented with 10% fetal bovine serum at 37°C in a humidified atmosphere containing 5% CO_2_.

### 2.9 Antibodies, siRNAs and reagents

Rabbit DCLRE1B antibody and mouse Homer1 antibody were purchased from omnimabs Co., Ltd (Alhambra, USA). Mouse β-actin antibody was purchased from ProMab Co., Ltd (California, USA). DCLRE1B siRNA and Homer1 siRNA were purchased from Shanghai GenePharma Co., Ltd (Shanghai, China). GP-transfect-Mate transfection kit was purchased from Shanghai GenePharma Co., Ltd (Shanghai, China). siRNAs targeting DCLRE1B and HOMER1 and their siRNA negative controls were transfected into A549 cells according to the manufacturer’s instructions by using GP-transfect-Mate transfection kit. siRNAs targeting DCLRE1B (siDCLRE1B-1: 5’CCAUAUGGAGAUCUGCCAUTT3’, siDCLRE1B-2: 5’CCGGACUCUGUACAGCAAUTT3’ and siDCLRE1B-3: 5’GGAUCAAGAAGCAGUUGUUTT3’). siRNAs targeting HOMER1 (siHOMER1-1: 5’GCAUCAUCUUUCGAAAUUUTT3’, siHOMER1-2: 5’GGUACCCACCAGCAAGCAUTT3’ and siHOMER1-3: 5’GCACUCGAGCUCAUGUCUUTT3’).

### 2.10 RT-qPCR and western blot

2 ug RNA was reverse transcribed using RevertAid-TM M-MuLV Reverse Transcription kit according to the manufacturer’s instructions and then cDNA was restored in -20°C. RT-qPCR reaction conditions were 94 °C for 20 s followed by 40 cycles of 72 °C for 20 s and 55°C for 10 s. RT-qPCR primers for DCLRE1B (forward: 5’GACCCACCCTACGATTGCTA3’, revers: 5’AGACTGTCCTGAAAGCCTCC3’). RT-qPCR primers for HOMER1 (forward: 5’GCACTCGAGCTCATGTCTTC3’, reverse: 5’CCACTGGCCAAACTTCTGAG3’). RT-qPCR primers for β-actin (forward: 5’CATTAAGGAGAAGCTGTGCT3’, revers: 5’GTTGAAGGTAGTTTCGTGGA3’). Western blot were performed according to our previous protocol (27, 28).

### 2.11 Cell proliferation assay, wound healing assay and transwell assay

Cell proliferation assay, wound healing assay and transwell assay were also carried out as we previously described (27, 28).

### 2.12 Statistical analysis

R software (v4.1.3) and GraphPad Prism software (v8.0.1) were utilized to perform statistical analyses and visualization. Student’s two-tailed t-test was used to compare the differences between distinct LUAD subtypes. Kaplan–Meier survival analysis and the log-rank test were used to compare the differences in survival time. All *P* values were two-sided, and a *P*< 0.05 was considered statistically significant unless otherwise stated.

**Table 1:** Clinical features of patients in subtypes 1 and 2.

Name	Clinical features	Subtype 1 (N=231)	Subtype 2 (N=100)	Total (N=331)	*P*
OS	Alive	176 (76.2%)	57 (57%)	233 (70.4%)	< 0.001
	Dead	55 (23.8%)	43 (43%)	98 (29.6%)	
Age	<= 65	107 (46.3%)	54 (54%)	161 (48.6%)	0.244
	> 65	124 (53.7%)	46 (46%)	170 (51.4%)	
Gender	Female	125 (54.1%)	42 (42%)	167 (50.5%)	0.043
	Male	106 (45.9%)	58 (58%)	164 (49.5%)	
Stage	I	130 (56.3%)	44 (44%)	174 (52.6%)	0.007
	II	56 (24.2%)	22 (22%)	78 (23.6%)	
	III	33 (14.3%)	25 (25%)	58 (17.5%)	
	IV	12 (5.2%)	9 (9%)	21 (6.3%)	
T	T1	76 (32.9%)	22 (22%)	98 (29.6%)	0.023
	T2	127 (55%)	62 (62%)	189 (57.1%)	
	T3	17 (7.4%)	11 (11%)	28 (8.5%)	
	T4	11 (4.8%)	5 (5%)	16 (4.8%)	
N	N0	158 (68.4%)	55 (55%)	213 (64.4%)	0.045
	N1	43 (18.6%)	24 (24%)	67 (20.2%)	
	N2	29 (12.6%)	21 (21%)	50 (15.1%)	
	N3	1 (0.4%)	0 (0%)	1 (0.3%)	
M	M0	219 (94.8%)	91 (91%)	310 (93.7%)	0.29
	M1	12 (5.2%)	9 (9%)	21 (6.3%)	

## 3 Results

### 3.1 Landscape of TPRGs and gene mutations

**Figure 1:**
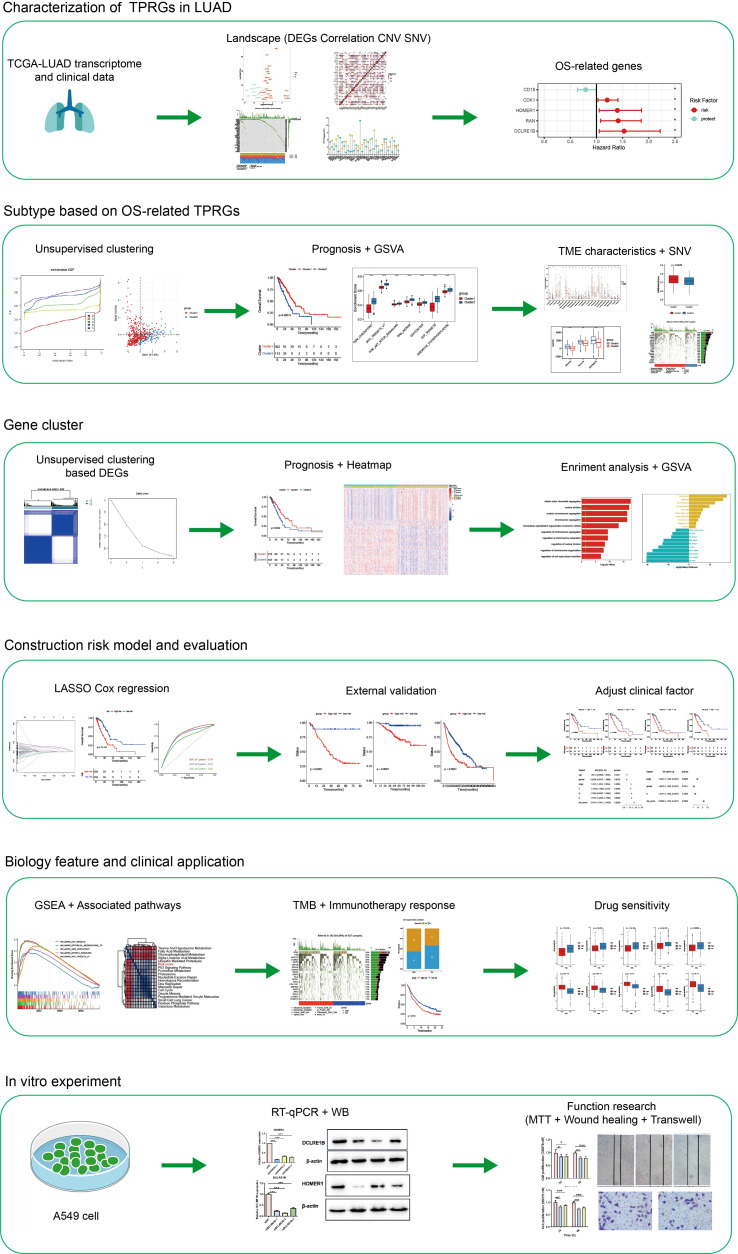
The flow chart.

**Figure 2:**
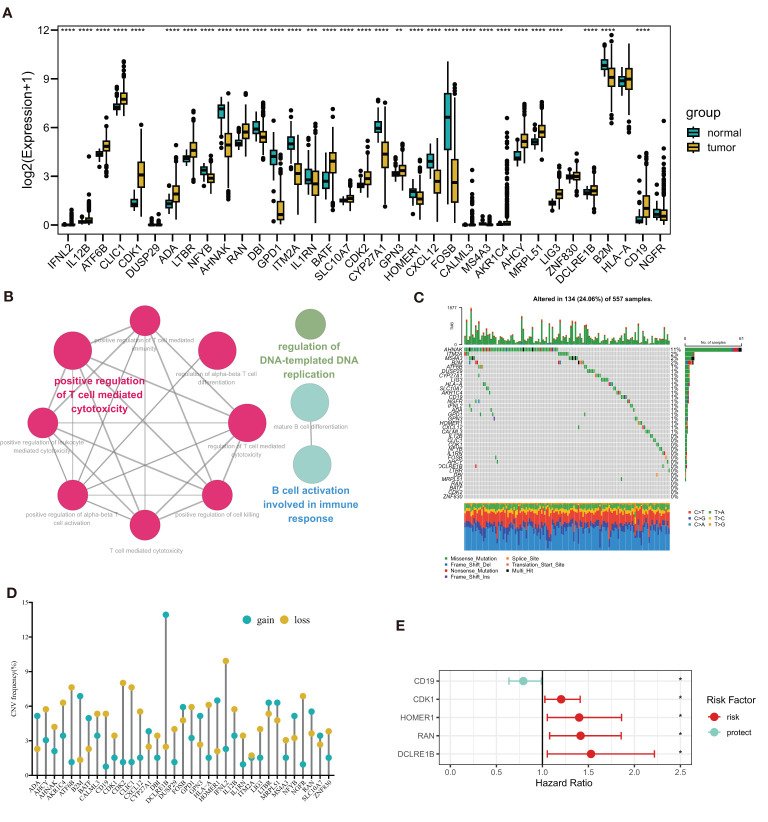
Landscape of T cell proliferation-related regulator genes (TPRGs) in LUAD. **(A)** Differentially expressed TPRGs in normal tissue and tumor tissue. **(B)** Potential biofunctional network associated with TPRGs from the STRING platform. **(C)** Mutation waterfall plots of 557 LUAD patients from the TCGA-LUAD cohort. **(D)** Copy number variation (CNV) frequency of TPRGs in the TCGA-LUAD cohort. **(E)** Hazard ratios (HR) forest plot of 5 TPRGs with prognostic values. HR > 1 (red) represents risk factors. HR (blue)< 1 represents protection factors. *P < 0.05, **P < 0.01, ***P < 0.001 and ****P < 0.0001.

In **Figure 1**, a flow chart of this study is showed. Transcription profile of TCGA-LUAD dataset was uesd for exploring the expression of the TPRGs. We found that there were 19 up-regulated TRPGs and 12 down-regulated TRPGs in the tumors in contrast to adjacent normal tissues (**Figure 2A**). STRING platform was used to analyze the potential biofunctional network associated with TPRGs (**Figure 2B**). Then, we explored the incidence of somatic mutations and CNVs for the TPRGs. The result showed that 134 of 557 patients (24.06%) have genetic alterations in TPRGs. Among them, *AHNAK* had the highest mutation frequency (11%), followed by ITM2A (2%) and MS4A3 (2%) (**Figure 2C**). The result of CNVs incidence showed that CNV alteration was prevalent in all TPRGs. Among them, B2M, DCLRE1B, MOMER1 showed remarkable copy number amplification, while ATF6B, CD19, CDK2, CLIC1, CXCL12, HLA-A, IFNL2 and NGFR showed significant copy number deletions (**Figure 2D**). We utilized univariate cox analysis to explore relevance of TPRGs with prognosis. Forest plot showed that CD19 was a protective factor, while CDK1, HOMER1, RAN and DCLRE1B were risk factors (**Figure 2E**).

### 3.2 Subtype classification based on OS-related TPRGs and enrichment analysis

**Figure 3:**
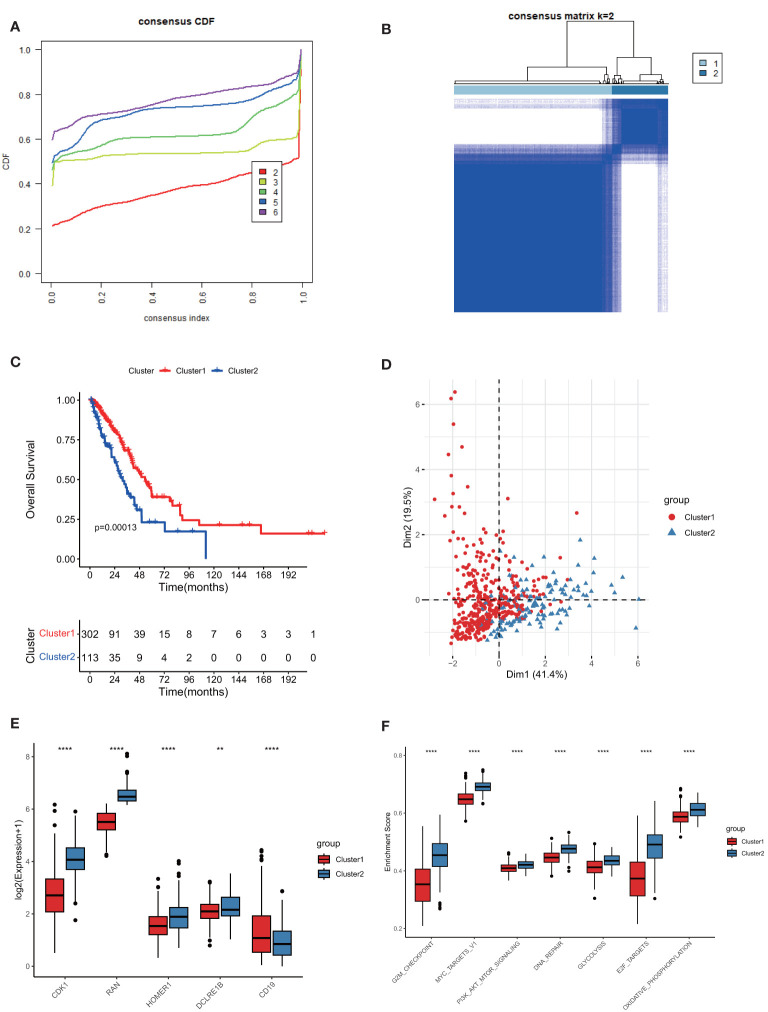
Subtype classification based on 5 prognostic T cell proliferation-related regulator genes (TPRGs) and enrichment analysis. **(A)** Consensus clusters by TPRGs. **(B)** Clustering heatmap. Consensus matrix at optimal k = 2. GCTA-LUAD patients were classified into clusters 1 and 2. **(C)** Overall survival analysis for the clusters 1 and 2. **(D)** PCA analysis. **(E)** Expression of five risk-related TPRGs (including CDK1, RAN, HOMER1, DCLRE1B and CD19) in the two clusters. **(F)** Enrichment analysis of proto-oncogene carcinogenic pathways in the two clusters. ** *P*< 0.01 and **** *P*< 0.0001.

We performed a consensus clustering analysis based on those 5 prognostic TPRGs (CD19, CDK1, HOMER1, RAN and DCLRE1B). By using cumulative distribution function (CDF), we divided LUAD into 2 TPRGs-associated subtypes (clusters 1 and 2) (**Figures 3A, B** and **Table 1**). When k = 2, the change of CDF value was relatively smooth (**Figure 3A**), and the heatmap of consensus matrix was relatively distinct (**Figure 3B**). Kaplan–Meier curve showed that the cluster 1 tended to have a longer OS (**Figure 3C**). The PCA algorithm also demonstrated that the cluster 1 was clearly separated from the cluster 2 (**Figure 3D**). Combining these results, we confirmed that the optimal cut-off of k-value was 2, and obtained two TPRGs-related subtypes. We also compared the expressions of the 5 prognostic TPRGs in the two clusters. A higher expression of protective factor CD19 and lower expressions of risk factors (including CDK1, DCLRE1B, HOMER1 and RAN) were found in the cluster 1 (**Figure 3E**). We further analyzed the enrichment score of cancer-related pathway in the two clusters. Our analysis result revealed that cluster 1 had a significantly lower proto-oncogene carcinogenic pathway activity than cluster 2 (**Figure 3F**).

**Figure 4:**
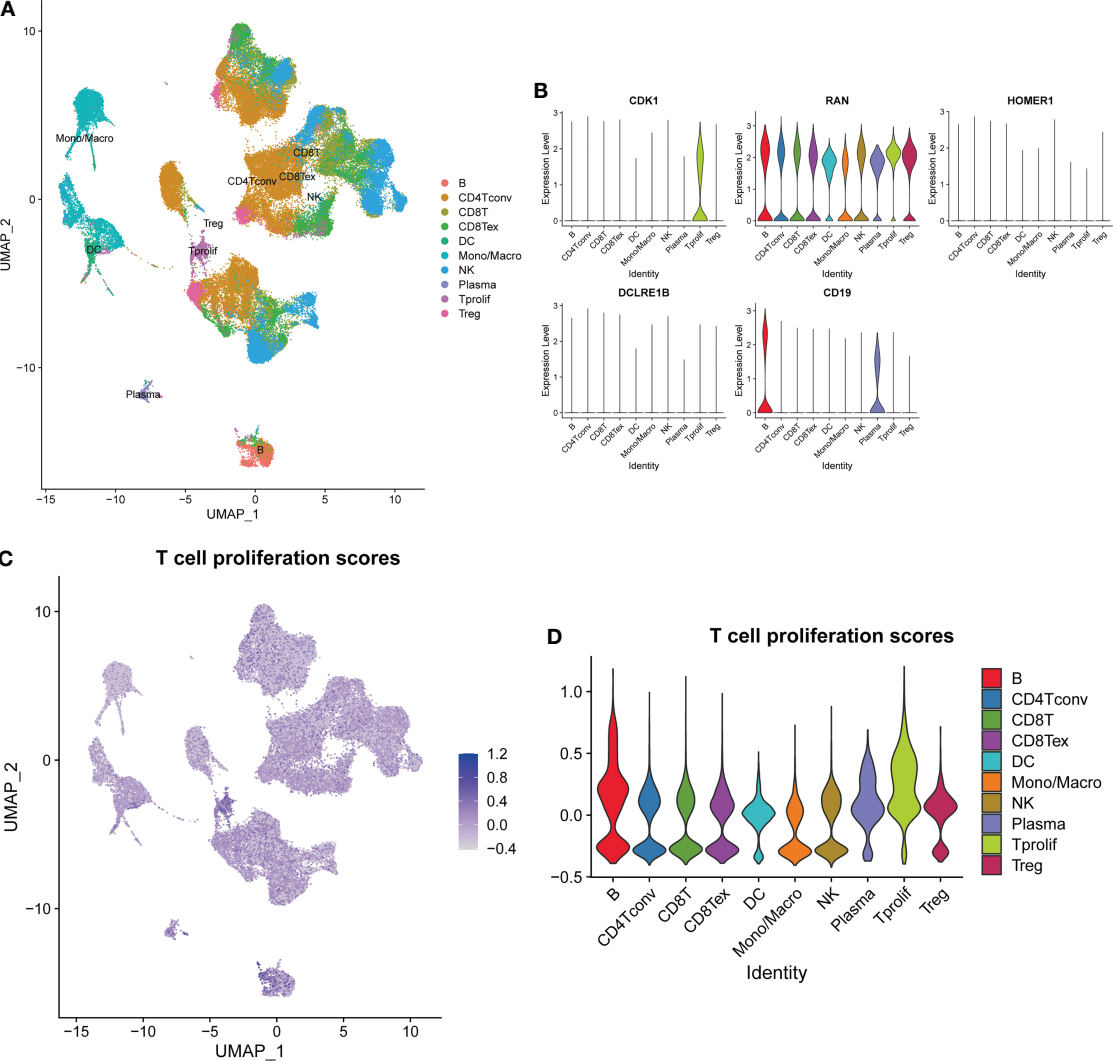
Overview of single cells from lung adenocarcinoma tissues and normal tissues. **(A)** Distributions of ten types of immune cell in tissues. **(B)** Expression profiles of 5 prognostic T cell proliferation-related regulator genes in the ten major types of immune cells. **(C)** The deeper the color, the higher the T cell proliferation score. **(D)** T cell proliferation scores in the ten major types of immune cells.

To further explore expression profiles of the TPRGs in TME of LUAD, we performed single-cell RNA sequencing analyses and clustering using a single-cell dataset GSE139555. Distributions of ten types of immune cells (including B, CD4+ T conv, CD8+ T, CD8+ Tex, DC, Mono/Macro, NK, plasma, T prolif and Treg) were identified in **Figure 4A**. Among those immune cells, T prolif cells had the highest T cell proliferation scores based on the expression levels of TRPGs, followed by B cells and plasma cells (**Figures 4C, D**). In addition, we measured expressions of the 5 prognostic TPRGs (including CDK1, DCLRE1B, HOMER1 and RAN) in the ten types of immune cell (**Figure 4B**).

### 3.3 Characterization of TME cell infiltration and gene mutation

**Figure 5:**
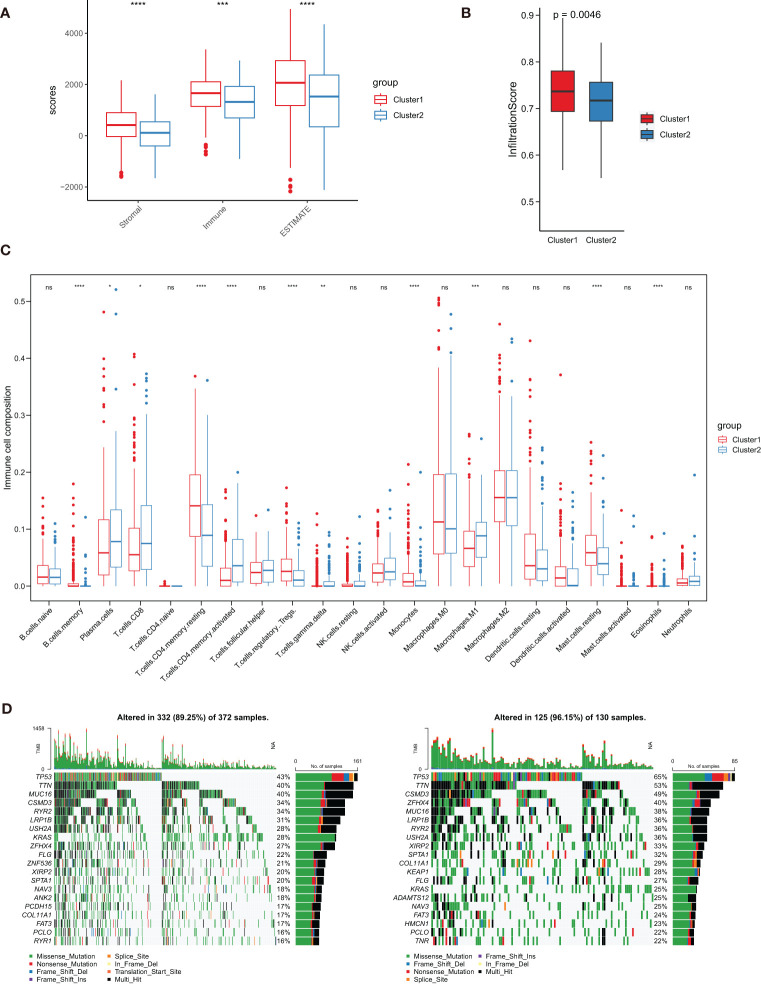
Characterization of tumor microenvironment (TME) cell infiltration and somatic mutation in clusters 1 and 2. **(A)** Analysis of differences in TME scores (including Stromal score, Immune score and ESTIMATE score). **(B)** Analysis of differences in Infiltration score. **(C)** Boxplot shows the infiltration abundance of 22 immune cells obtained from CIBERSORT analysis. **(D, E)** Comparison of the top 20 genes with the highest somatic mutation frequency in the two clusters. ∗ *P*< 0.05, ∗∗ *P*< 0.01, ∗∗∗ *P*< 0.001, ∗∗∗∗ *P*< 0.0001 and ns represents nonsense.

To better understand the interaction between TPRGs and TME, we investigated the immune cell infiltration in clusters 1 and 2. First, we used ESTIMATE method to calculate the Stromal score, Immune score and ESTIMATE score in the two clusters, and found the cluster 1 had higher infiltration than the cluster 2 (**Figure 5A**). We also utilized the ImmuCellAI to obtain the Infiltration scores in LUAD and the result also showed that the cluster 1 had a higher level of immune infiltration than the cluster 2 (**Figure 5B**). Furthermore, cell infiltrations of 22 immune cells in TME were compared using CIBERSORT. The two clusters showed distinct immune infiltration patterns that eleven types of immune cells were significantly differently infiltrated (**Figure 5C**). Moreover, we investigated and compared the incidence of somatic mutations between clusters 1 and 2. For the cluster 1, 332 of 372 patients (89.25%) had somatic mutations (**Figure 5D**). For the cluster 2, 125 of 130 samples (96.15%) had somatic mutations (**Figure 5D**). The cluster 1 patients had lower somatic mutation frequencies in some important anti-oncogenes (for example, TP53 and KRAS) than the cluster 2 patients (**Figure 5D**).

### 3.4 Gene clustering

**Figure 6:**
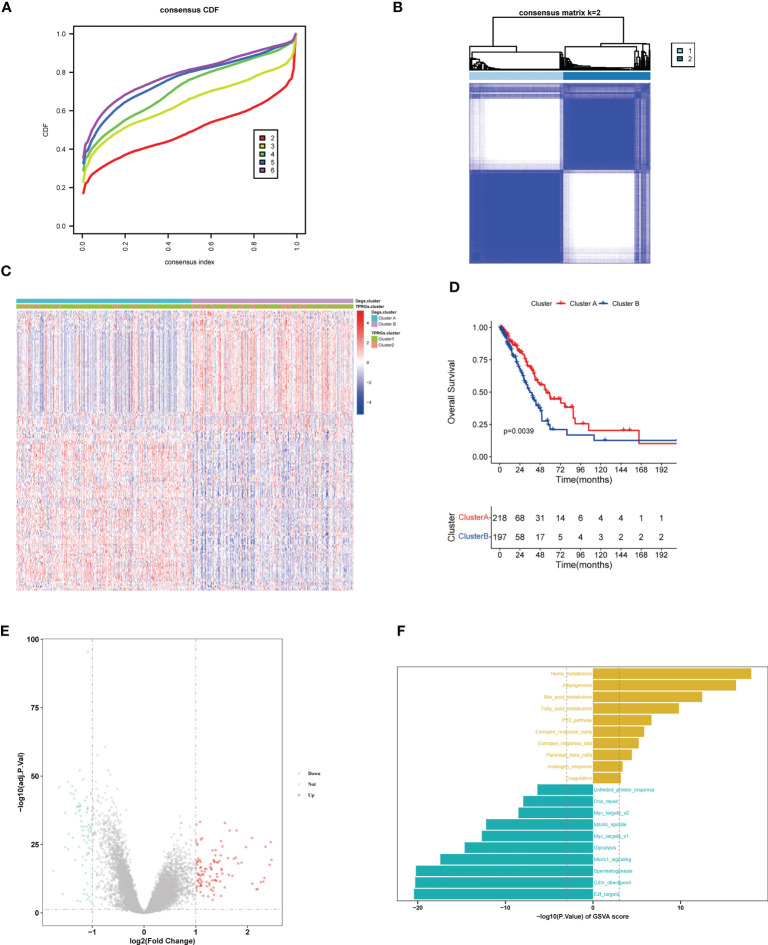
LUAD clustering (cluster **A** and cluster **B**) based on differentially expressed genes (DEGs) between clusters 1 and 2. **(A)** Identification of consensus clusters (clusters **A** and **B**) based on DEGs between cluster 1 and cluster 2. **(B)** Clustering heatmap of clusters **(A, B)** Consensus k=2. **(C)** Heatmap shows the DEGs expression profiles in the different LUAD clusters (including cluster 1, cluster 2, cluster **A** and cluster **B**). **(D)** Kaplan–Meier survival curves of overall survival in the clusters **(A, B, E)** Volcano plot for DEGs analysis between clusters **(A, B, F)** Gene Set Variation Analysis of T cell proliferation-related regulator genes in clusters **(A, B)**.

By performing R package “limma”, we found 103 up-regulated genes and 77 down-regulated genes between the two TPRGs-related clusters. Based on the expression profiles of these dysregulated genes, we conducted an unsupervised clustering analysis. K = 2 was the optimal number for clustering with the distinct heatmap (**Figures 6A, B**). As shown in heatmap (**Figure 6C**), two subtypes (clusters A and B) had significantly distinct expression profiles. The resemblance between cluster A and cluster 1, or cluster B and cluster 2 was remarkable according to DEGs expression heatmap (**Figure 6C**). Moreover, similarly with the OS in the clusters 1 and 2 (**Figure 3C**), the cluster A had longer OS than the cluster B (**Figure 6D**). Subsequently, we compared DEGs between the clusters A and B (**Figure 6E**). To explore potential biological function and pathway of the DEGs, we compared 50 hallmark pathways using GSVA. Up-regulated hallmark gene sets in the cluster A were mainly enriched in metabolisms (including heme metabolism, adipogenesis, bile acid metabolism and fatty acid metabolism) and p53, while the up-regulated hallmark gene sets in the cluster B were mainly enriched in the e2f targets, G2m checkpoint, spermatogenesis and mTORC1 signaling (**Figure 6F**).

### 3.5 Construction of a predictive risk model

**Figure 7:**
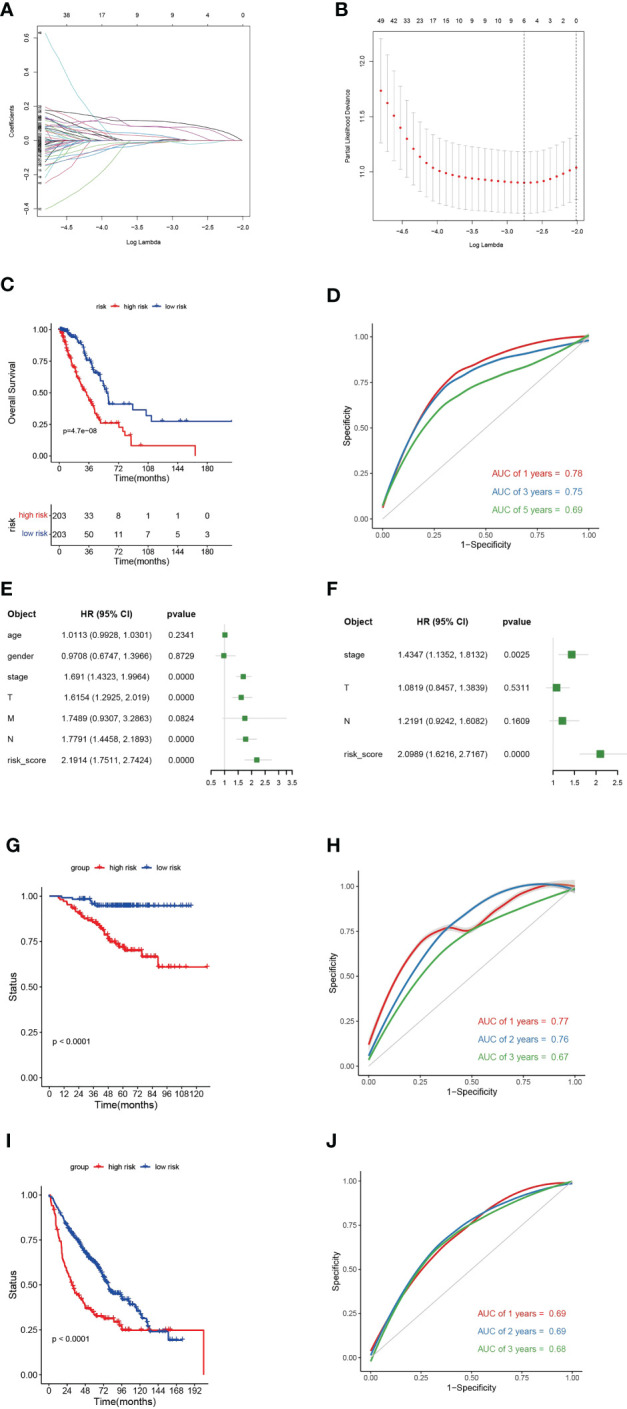
A 6-gene risk model based on differentially expressed genes (DEGs) between clusters **(A)** and **(B, A)** Partial likelihood deviance coefficient profiles. **(B)** Least absolute shrinkage and selection operator cox analysis of the DEGs between clusters A and **(B, C)** Kaplan-Meier curves for overall survival (OS) of the high- and low-risk subtypes in TCGA-LUAD cohort. **(D)** Receiver operating characteristic (ROC) curves for 1-, 2- and 3-year of TCGA-LUAD cohort. **(E)** Multivariate cox regression analyses show the hazard ratios of 6-gene risk model and other clinic-pathological factors. **(F)** Univariate cox regression analyses show the hazard ratios of the risk model and other clinic-pathological factors. **(G)** Kaplan-Meier curves for OS of the high- and low-risk subtypes in GEO-LUAD cohort (GSE31210). **(H)** ROC curves for 1-, 2- and 3-year of GEO-LUAD cohort (GSE31210). **(I)** Kaplan-Meier curves for OS of the high- and low-risk subtypes in GEO-LUAD cohort (GSE68465). **(J)** ROC curves for 1-, 2- and 3-year of GEO-LUAD cohort (GSE68465).


We first identified TPRGs-related differentially expressed genes (DEGs) between gene cluster A and gene cluster B. Then, we conducted a univariate Cox regression analysis to obtain DEGs with prognostic value according to the threshold of *P* value< 0.05. At last, we constructed the 6-gene signature using the LASSO Cox model with the parameters of 10-fold cross-validation and 1000 reps (**Figures 7A, B**). The risk score of each LUAD patient according to the formula:

$$\begin{array}{l} \text{Riskscores}=0.00053*\text{Exp CCNA}2+0.04793*\text{Exp HMMR}+\\ 0.07896*\text{Exp ANLN}+\left(-0.02199\right)*\text{Exp NKX}2-1+\\ \left(-0.00044\right)*\text{Exp SFTPB}+ð-0.0938*\text{Exp KRT}6\text{A}. \end{array}$$

The 6-gene risk model divided TCGA-LUAD patients into high- and low-risk subtypes based on the median risk scores. The high-risk subtype had a significantly worse clinical outcome than the low-risk subtype (**Figure 7C**). The 6-gene risk model showed a good sensitivity and specificity in stratifying TCGA-LUAD patients using ROC curves (for 1-year, areas under the curve (AUC) = 0.78; for 2-year, AUC = 0.75; for 3-year, AUC = 0.69) (**Figure 7D**). To determine whether the 6-gene risk model was an independent factor, we combined muinltivariate and univariate Cox regression analyses. The forest plot showed that the 6-gene risk model was an independent risk factor even when combined with various clinical features (**Figures 7E, F**). The predictive ability of the 6-gene risk model was further validated in two external GEO cohorts (GSE31210 and GSE68465) (**Figures 7G–J**). In both of the two GEO datasets, Kaplan-Meier curves showed significant shorter OS for the high-risk subtype (**Figures 7G, I**). For the GSE31210, the AUCs at 1-, 3- and 5-year were 0.77, 0.76 and 0.67, respectively (**Figure 7H**). For the GSE68465, the AUCs at 1-, 3- and 5-year were 0.69, 0.69 and 0.68, respectively (**Figure 7J**). Collectively, those results verified that the TPRGs-related risk model classified the patients well and showed a good sensitivity and specificity.

### 3.6 Associations of the 6-gene risk model with clinical features

**Figure 8:**
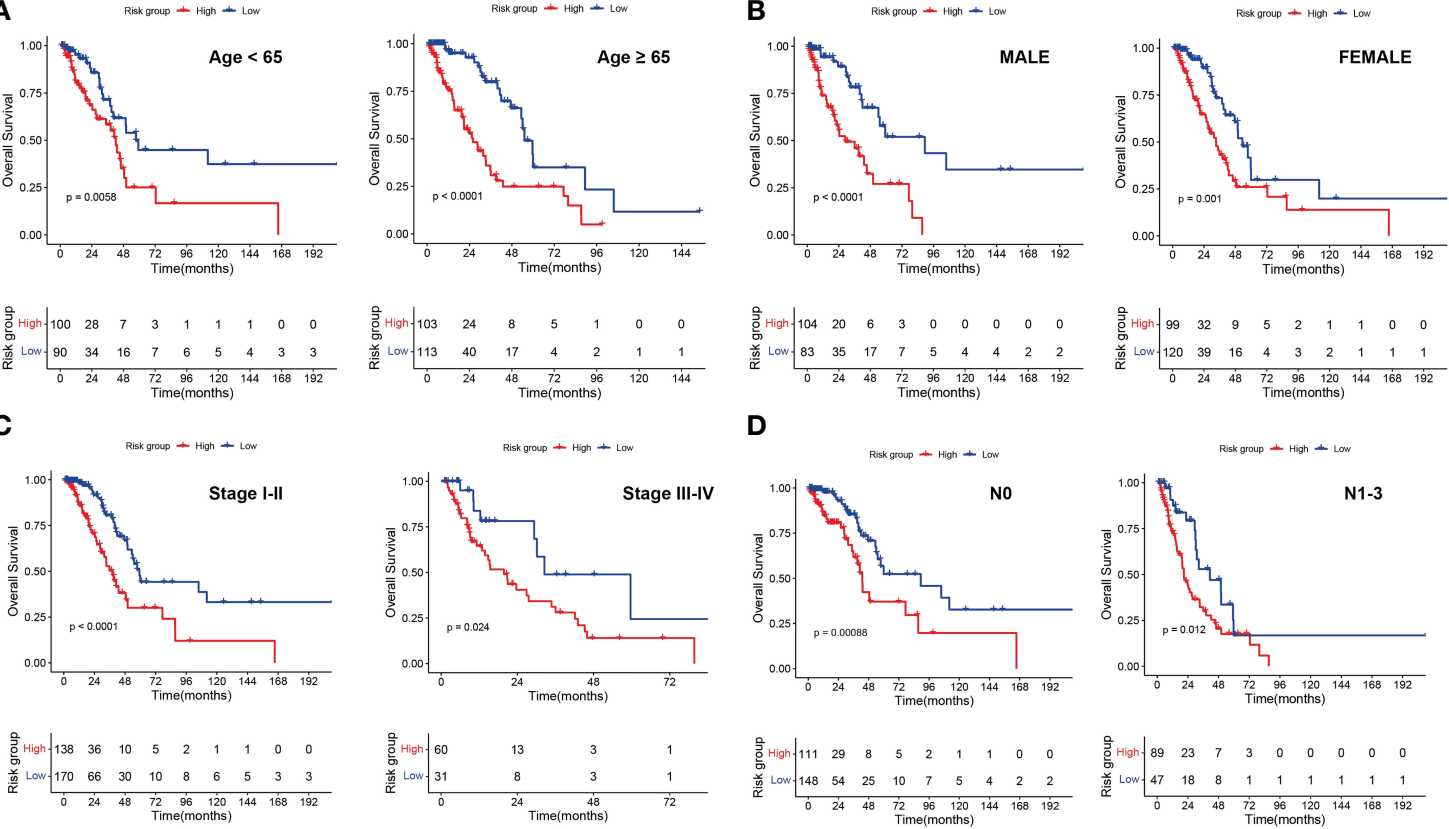
Associations of risk scores with clinical characteristics by stratification analyses. Kaplan‑Meier survival curves for overall survival of LUAD patients stratified by age **(A)**, gender **(B)**, clinical stage **(C)** and N stage **(D)**.

To investigate the relevance between risk model and other clinical variables, we performed survival analyses according to various clinical parameters (including age (≥ 65/>65), gender (female/male), stage (I-II/III-IV) and N (0/1-3)). In each stratum of the above clinical features, the high-risk subtype had significant worse survival outcome than the low-risk subtype (**Figure 8**). These results demonstrated that our risk model still had a reliable ability to predict OS within each stratum and could be applicable for LUAD patients stratified by different clinical parameters.

**Figure 9:**
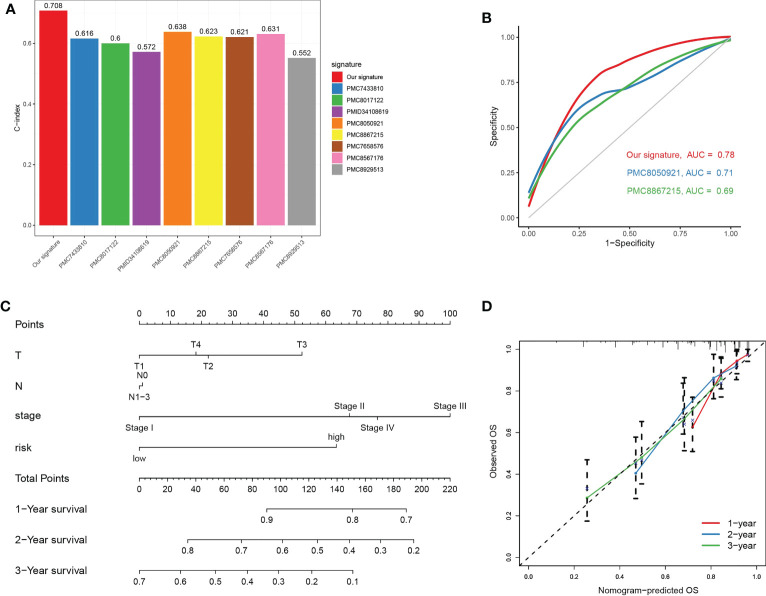
Prognostic nomogram combing key clinical features. **(A)** Comparison of C-Index between our TPRGs risk model and a few published models (including PMC7433810, PMC8017122, PMID34108619, PMC8050921, PMC8867215, PMC7658576, PMC8567176 and PMC8929513). **(B)** Receiver operating characteristic curves for 1-year using our risk model and two other published models (PMC8050921 and PMC8867215). **(C)** A nomogram combining T, N, stage and risk score predicts 1-, 3-, and 5-years survival. **(D)** Calibration curves test the agreement between observed and predicted overall survival at 1-, 3-, and 5-year.

We compared C-index of our risk model with a few published models (including PMC7433810, PMC8017122, PMID34108619, PMC8050921, PMC8867215, PMC7658576, PMC8567176 and PMC8929513), and our TGPGs risk model had the highest C-index (**Figure 9A**). Then we compared accuracy of our risk model and two risk models (PMC8050921 and PMC8867215) with relatively higher C-index. ROC curves for 1-year showed that our risk model had a better accuracy than the other two risk models (for our risk model, AUC = 0.78; for PMC8050921, AUC = 0.71; for PMC8867215, AUC = 0.69) (**Figure 9B**). Moreover, we constructed a prognostic nomogram using factors using T, N, stage and risk score (**Figure 9C**). The nomogram could reliably predict 1-, 2-, and 3-year OS of LUAD patients (**Figure 9D**).

### 3.7 Function and pathway enrichment analyses

**Figure 10:**
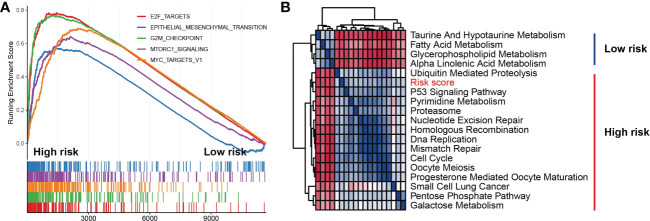
Function and pathway enrichment analyses in high- and low-risk subtypes. **(A)** Gene set enrichment analysis of the top 5 gene hallmarks significantly enriched in high-risk subtype. **(B)** Kyoto encyclopedia of genes enrichment analysis of high-risk subtype and low-risk subtype.

To explore the association of risk scores to biological behaviors, functional annotations of TCGA-LUAD was used for the high- and low-risk subtypes. The top 5 gene hallmarks of Gene Set Enrichment Analysis (GESA) and 18 Kyoto Encyclopedia of Genes (KEGG) pathways with |correlation| > 0.3 & p< 0.05 were visualized (**Figure 10**). For the high-risk subtype, the GESA result was enriched in hallmark e2f targets, hallmark epithelial mesenchymal transition, hallmark G2M checkpoint, hallmark mTORC1 signaling and hallmark myc targets v1 (**Figure 10A**). In **Figure 10B**, the correlation heatmap showed that the low-risk subtype was mainly enriched in metabolic-related functions and pathways (including taurine and hypotaurine metabolism, fatty acid metabolism, glycerophospholipid metabolism and alpha linolenic acid metabolism), while the high-risk subtype was mainly enriched in tumor-related pathways and functions (for instance, p53 signaling pathway, nucleotide excision repair, homologous recombination, DNA replication, mismatch repair, cell cycle and small cell lung cancer).

### 3.8 Somatic mutation frequency and predictability of immunotherapy response

**Figure 11:**
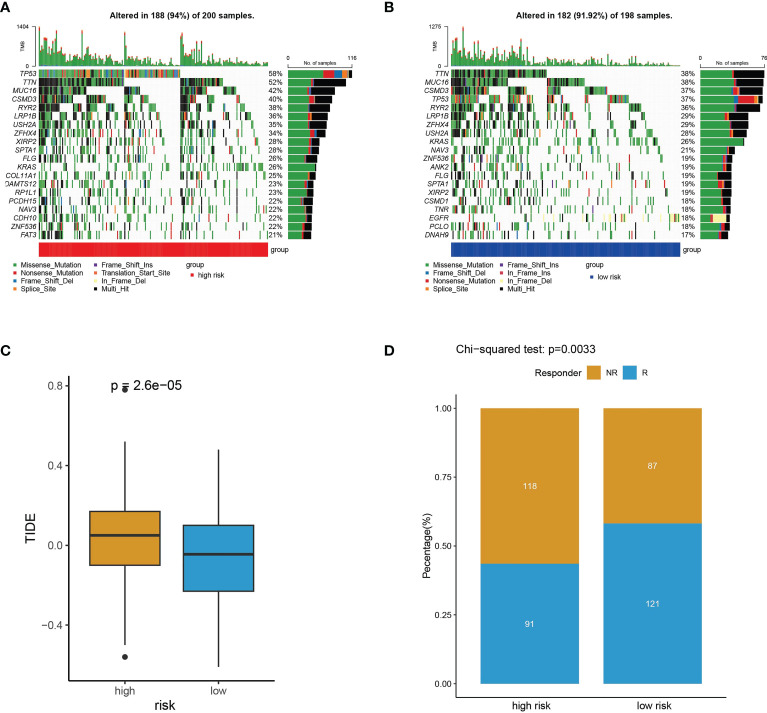
Somatic mutation frequency and immunotherapy response analyses. **(A, B)** The top 20 genes with the highest somatic mutation frequency in high-risk subtype **(A)** and low-risk subtype **(B)**. **(C)** TIDE prediction scores for immunotherapy response in the high- and low-risk subtypes. **(D)** The difference of high- and low-risk subtypes between non-responders (NR) and responders (R).

To explore the relevance of the risk model and somatic mutation in TCGA-LUAD, we counted incidence of genetic alterations in the high- and low-risk subtypes. For the high-risk subtype, 188 of 200 samples (94%) had genetic alterations (**Figure 11A**). For the low-risk subtype, 182 of 198 samples (91.92%) had genetic alterations (**Figure 11B**). Compared with the low-risk subtype, the high-risk subtype had higher genetic alteration frequencies in TP53, TTN, MUC16, LRP1B, ZFHX4 and USH2A (**Figures 11A, B**). Somatic mutation burden has been widely described as a biomarker for response to immune checkpoint inhibitors (29–32). Because of the obviously differences in somatic mutation frequency between the high- and low-risk subtypes, we used the TIDE algorithm (25) to explore if the risk scores could reflect the immunotherapeutic benefit in LUAD patients. The boxplot indicated that the high-risk subtype had higher TIDE scores (**Figure 11C**) and a significant lower immunotherapy responder number (91/209) than the low-risk subtype (121/209) (chi-square tests, p = 0.0033) (**Figure 11D**). The high-risk subtype characterized by higher somatic mutation frequency and lower immunotherapy response had a worse prognosis (**Figures 7**, **11**).

### 3.9 Drug sensitivity analyses

**Figure 12:**
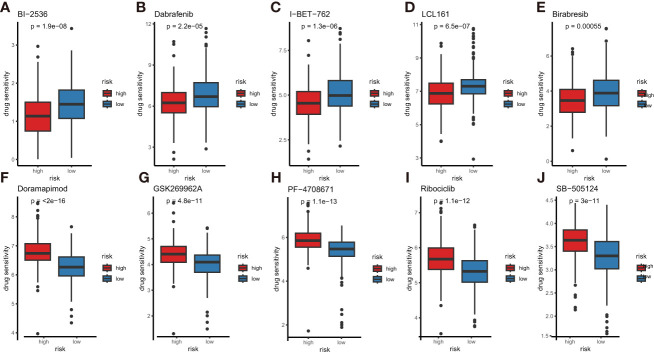
Comparison of anti-tumor drug sensitivity between the high- and low-risk subtypes. **(A)** BI-2536, **(B)** dabrafenib, **(C)** I-BET-762, **(D)** LCL-161, **(E)** Birabresib, **(F)** doramapimod, **(G)** GSK269962A, **(H)** PF-4708671, **(I)** ribociclib, **(J)** SB-505124.

To help doctors make better drug treatments for different risk groups, we compared the half-maximal inhibitory concentration (IC50) values of drugs between the high- and low-risk subtypes by performing the oncoPredict package. The results indicated that Dabrafenib, Birabresib, I-BET-762, BI-2536 and LCL-161 had higher IC50 in the low-risk subtype, suggesting that these drugs are more resistant in the LUAD patients with low risk (**Figures 12A–E**). In contrast to that, the IC50 values of Ribociclib, Doramapimod, GSK269962A, PF-4708671 and SB-505124 were higher for the high-risk subtype, indicating that these drugs are more effective in the patients with low-risk scores (**Figures 12F–J**). These drug sensitivity analyses might guide individualized treatment strategies.

### 3.10 Functional validation of DCLRE1B and HOMER1

**Figure 13:**
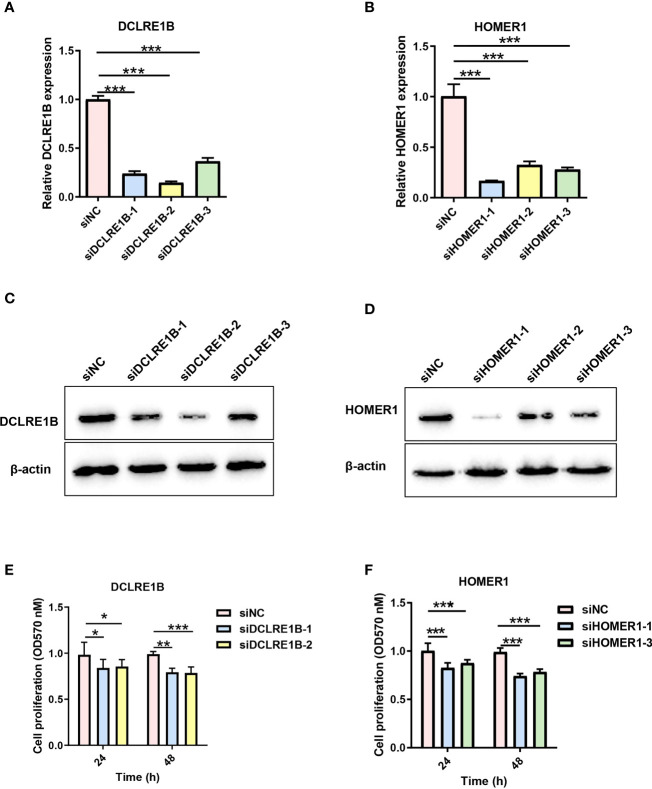
Validation of cell proliferation for DCLRE1B and HOMER1 in LUAD cell line A549. A549 cells were transiently transfected with siRNA (siDCLRE1B-1, siDCLRE1B-2, siDCLRE1B-3, siHOMER1-1, siHOMER1-2 or siHOMER1-3) or with its corresponding negative control (siNC). RT-qPCR **(A, B)** and western blot **(C, D)** were used to measure DCLRE1B and HOMER1 expressions.**(E, F)** MTT assay was used to measure cell proliferation. The data were presented as the mean ± standard deviation. ∗ *P*< 0.05, ∗∗ *P*< 0.01 and ∗∗∗ *P*< 0.001.

**Figure 14:**
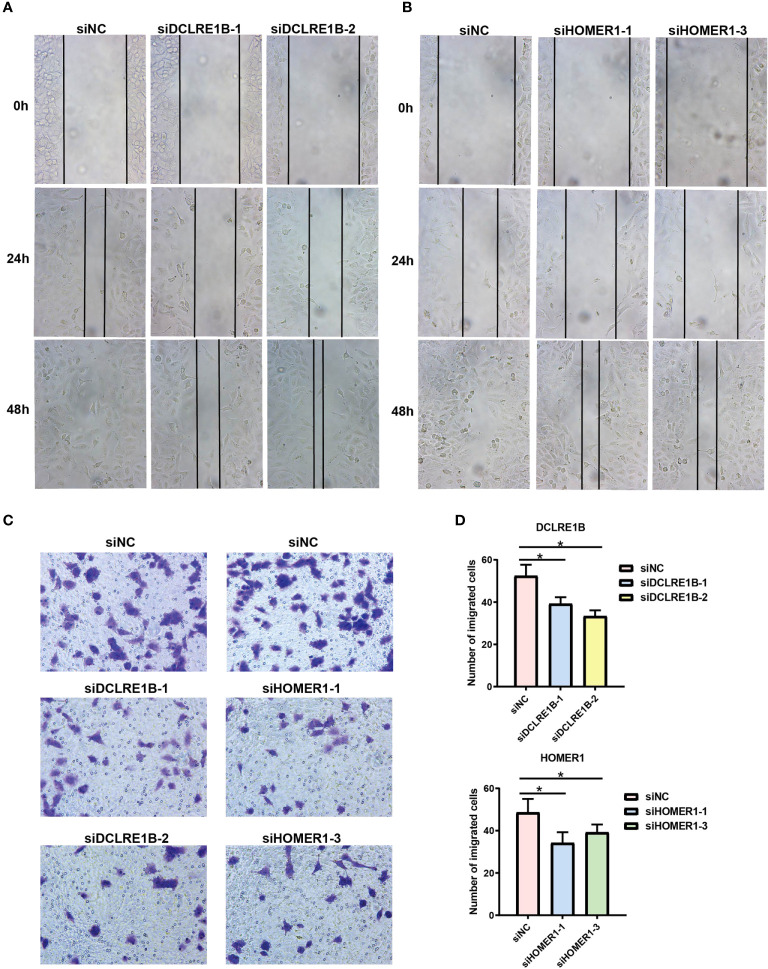
Validation of cell migration and invasion for DCLRE1B and HOMER1 in LUAD cell line A549. A549 cells were transiently transfected with siRNAs (siDCLRE1B-1, siDCLRE1B-2, siHOMER1-1 or siHOMER1-3) or with its corresponding negative control (siNC). **(A, B)** Wound healing assay was used to measure cell migration. **(C, D)** Transwell assay was used to measure cell invasion. Representative images were shown on the right. The data were presented as the mean ± standard deviation. ∗ *P*< 0.05.

According to our prognostic value analysis, DCLRE1B and HOMER1 were risk factors in LUAD (**Figure 2D**). Currently, their cellular effects in lung cancer are unclear. To validate the cellular effects of HOMER1 and DCLRE1B, we performed MTT, wound healing and transwell assays in LUAD cells A549 (**Figures 13**, **14**). As showed in **Figure 13**, DCLRE1B could be knocked down by three siRNAs (siDCLRE1B-1, siDCLRE1B-2 and siDCLRE1B-3) and HOMER1 also could be scilenced by thee siRNAs (siHOMER1-1, siHOMER1-2 and siHOMER1-3) at both mRNA (**Figure 13A, B**) and protein (**Figure 13C, D**) levels. The siDCLRE1B-1, siDCLRE1B-2, siHOMER1-1 and siHOMER1-3 were chosen as optimal siRNAs for further experiments. After transiently transfected with siRNAs, significant decreases in cell proliferation (**Figure 13E, F**), migration (**Figure 14A, B**) and invasion (**Figure 14C, D**) were observed in DCLRE1B- and HOMER1-silenced A549 cells compared with each control. These cellular effects of DCLRE1B and HOMER1 were in line with their prognostic values.

## 4 Discussions

The most commonly diagnosed lung cancer is NSCLC, of which is mainly LUAD. Recent advance in LUAD treatment targeting oncogenic drivers and immune checkpoints has shifted the paradigm of therapies, leading to a durable response and prolonged OS (33). However, tumor shrinkage or extended survival is still limited to a small portion of patients. Moreover, LUAD patients with similar AJCC/UICC-TNM features have distinct outcomes. It is getting clearer that the reason should be sought in the malignant cells and in the multiple interactions between malignant cells and TME (5). The composition of a tumor is not only a group of heterogeneous malignant cells, but also a TME that contains infiltrating immune cells, extracellular matrix molecules, etc. The TME differs across individual patients and will, in turn, determine tumor characteristics and patient outcomes (34). At present, TME represents an element of increasing interest for prognostic tool and a therapeutic target in different cancers including LUAD (5, 35). Classifying patients into subtypes and constructing a sensitive and accurate risk model based on TPRGs in tumor may contribute to predicting prognosis for LUAD.

Our current study aims to classify LUAD patients and construct a risk model for predicting outcomes. The risk model showed reliability and accuracy in outcome prediction. The 6-gene risk model can classify LUAD patients into distinct subtypes (high- and low-risk subtypes). The high-risk subtype was strongly associated with shorter OS of LUAD patients stratified by various clinical parameters. To explore the possible reasons, we performed functional and pathway enrichment analysis, somatic mutation frequency analysis and immunotherapy response analysis. Low-risk patients with longer OS were enriched in some important metabolic pathways (including fatty acid metabolism, heme metabolism, adipogenesis, bile acid metabolism, taurine and hypotaurine metabolism, glycerophospholipid metabolism and alpha linolenic acid metabolism), while the high-risk patients with shorter OS were enriched in oncogenic signaling pathways (including, E2f targets, G2m checkpoint, myc targets v1 and mTORC1). Oncogenic pathways in cancer cells can impair induction or execution of the local anticancer immune response (36). Oncogenic pathways also regulated immune checkpoint proteins and cancer immune surveillance, and ultimately favor resistance to immune checkpoint therapies (37). Thus, the activation of oncogenic pathways in high-risk patients may one important reason why high-risk patients had worse outcomes. High-risk subtype had higher genetic alteration frequencies in several important cancer-related genes, such as TP53, MUC16 and LRP1B. Around half of cancers have gene mutations in tumour suppressor Functional defect of TP53 results in carcinogenesis and drug resistance of cancer cells (38). Aberrantly expressed MUC16 is found in various cancers, which plays important roles in carcinogenesis, anti-cancer immune response and acquired resistance to drugs (39, 40). MUC16 is potential target for monoclonal antibodies and immunotherapy (39). Putative tumor suppressor LRP1B is frequently inactivated in cancers. LRP1B has been emerged as a a potential therapy target and associated with cancer responses to immune checkpoint inhibitor therapies (41). The results of immunotherapy response analyses showed that high-risk patients had higher TIDE scores and a significant lower immunotherapy responder number. Those results together suggested a less likely immunotherapeutic benefits for the high-risk patients. Drug efficacy or therapy is associated with drug sensitivity and individual variation. Thus individualized therapies based on subtypes will reduce ineffective treatments in LUAD patients. We compared the drug sensitivity between LUAD patients with different risk scores. Ten drugs with significantly differential sensitivity were found. The sensitivity prediction showed that Dabrafenib, Birabresib, I-BET-762, BI-2536 and LCL-161 were a better choice for high-risk patients, while Ribociclib, Doramapimod, GSK269962A, PF-4708671 and SB-505124 were more effective in low-risk patients. We notice that certain signaling pathways (for example, myc targets, e2f targets and mTORC1 signaling) by which some of those drugs exert their antitumor effects were enriched in our enrichment analyses (**Figure 3**, **6**, **9**). For example, I-BET-762 and Birabresib are bromodomain and extra-terminal inhibitors. I-BET-762 not only reduces cell proliferation and c-myc expression in NSCLC tumor but also altered immune populations in lung (42–44). NSCLC cells treated with Birabresib leads to myc down-regulation and cell proliferation inhibition (45). PF-4708671 stimulates S6K1 phosphorylation, which plays a key role in cell growth is dependent upon mTORC1 (46, 47). Ribociclib, a cyclin-dependent kinases CDK 4/6 inhibitor, has showed anti-tumor benefit in NSCLC (48). CDK 4/6 phosphorylate and inactivate retinoblastoma protein and subsequently negatively control e2f (49, 50). Cyclin D is a common downstream pathway for mTOR signaling (49).

Our results of single-cell RNA sequencing analyses and clustering showed that T prolif cells had the highest T cell proliferation scores based on the expression levels of TRPGs among ten types of immune cells. In the original study, most of these TPRGs have been demonstrated to increase the proliferation and activation of primary human CD4+ and CD8+ T cells and their secretion of key cytokines (17). TPRGs-related T cell proliferation can leads to better outcomes in LUAD patients, but doesn’t have to. T cells, the major tumor infiltrating immune cells of TME (51), comprises of various T cell subsets. T cell subsets and some other types of immune cells exert both tumor-antagonizing and tumor-promoting activities in the lung TME (52). For example, contrary to the immune-boosting functions of CD+8 T cells (15), Treg are well-known for their immune-suppressing activities (53). Moreover, in the TME, expanded T cell clones that do not recognize tumor cells are mentioned and, vice versa, small T cell clones present tumor inhibitory ability (15). It is noteworthy that not all of the TPRGs restrict to T cells. For example, TPRGs CDK1 and CXCL12 are expressed in both T cells and various cancer cells. A systematic pan-cancer analysis shows that oncogene CDK1 is an immunological and prognostic biomarker, which may influence tumor immunity mainly by mediating the migration of immune cells to TME, and is positively associated with tumor mutational burden and microsatellite instability (54). CXCL12 is highly enriched in fibroblasts (16). Fibroblasts in bladder cancer parietal tissue promote bladder carcinogenesis and progression by paracrine secretions of CXCL12 into TME to interact specifically with CXCR4 receptors (a specific receptor for CXCL12, expressed in T cells and macrophages in tumor tissues) and promote the proliferation of depleted T cells in cancer tissues (16). Those studies reveal complicated roles of TRPGs in cancer by regulating immune cells and cancer cells. Considering that functions of the TPRGs have already been explored in T cells (17), we further validated cellular effects of two TRPGs (including DCLRE1B and HOMER1) in LUAD cells. As a result, DCLRE1B and HOMER1 suppressed cell proliferation, migration and invasion, which line with their prognostic values (risk factors). The biological functions and underlying molecular mechanisms of those TRPGs need to be investigated in the future.

Overall, our study proposed a TPRGs-related 6-gene risk model for subtype classification and OS prediction in LUAD. The LUAD subtypes divided by the risk model showed remarkably differences in biology function and pathway, mutation status, immunity and drug susceptibility. High-risk subtype characterized by higher somatic mutation frequency and lower immunotherapy response had a shorter OS. The subtypes with different risk scores were significantly associated with drug sensitivity. Furthermore, TPRGs-encoded proteins DCLRE1B and HOMER1 suppressed cell proliferation, migration and invasion, which was in line with their prognostic values. This risk model showed a good reliability and accuracy in training and validation cohorts, and might serve as a potential prognostic biomarker in clinical use.

## Data availability statement

The original contributions presented in the study are included in the article/supplementary material. Further inquiries can be directed to the corresponding author.

## Author contributions

QY and HG contributed the idea for the article, performed the experiments and analyses, and wrote the manuscript. WZ revised the manuscript. All authors contributed to the article and approved the submitted version.
